# Acknowledgement to Reviewers of *Molecules* in 2013

**DOI:** 10.3390/molecules19032707

**Published:** 2014-02-25

**Authors:** Molecules Editorial Office

**Affiliations:** MDPI AG, Klybeckstrasse 64, CH-4057 Basel, Switzerland

The editors of *Molecules* would like to express their sincere gratitude to the following reviewers for assessing manuscripts in 2013:

Loboda, A.
Abaev, Vladimir
Abbotto, Abbotto
Abdel-Gawad, Fatma M.
Abdel-Megid, Mohamed
Abdelwhab, E. M.
Abdullah, Abdullah
Abonía, Rodrigo
Adam, Vojtech
Adiele, R. C.
Adireddy, Shiva
Afantitis, Antreas
Afonso, Carlos A. M.
Agarwal, Umesh P.
Agazzi, Agazzi
Agbor, Gabriel A.
Agbossou, Francine
Agrofoglio, Luigi
Ahmad, Ijaz
Ahmed, Mukhtar H.
Ahn, Joong-Hoon
Ahn, Sung-Hoon
Aires, Alfredo
Aisa, Haji Akber
Ait-Oudhia, Sihem
Akamatsu, Miki
Akhtar, Yasmin
Al-Tel, Taleb
Al. Marghitas, Liviu
Alam, M. S.
Alam, Todd
Albericio, Fernando
Albert, Joan
Alcântara, Ana C. S.
Alcaráz, Lucía Esther
Alché, Laura E.
Alebic-Kolbah, Tanja
Alemán, José
Antunes, Alexandra M. M.
Alezra, Valérie
Alhaique, Franco
Ali, Ihsan
Ali, Saqib
Alkharfy, Khalid M.
Allan, Andrew C.
Allison, Stuart A.
Almajano, Maria Pilar
Almeida, Wanda
Almendros, Pedro
Almerico, Anna Maria
Alonso, Diego A.
Alunda, José Ma
Alwan, Shakir Mahmood
Alway, Stephen E.
Amabilino, David B.
Amann, Anton
Amarowicz, Ryszard
Amat, Mercedes
Amboni, R. D. M. C.
Ambrosio, Sergio R.
Ambudkar, Suresh V.
Amin, Kawa
Anand, Sathanandam S.
Anderson, Carolyn
Anderson, J. P.
Andjelkovic, Anuska V.
Andrade, Carolina Horta
Andrade, Paula Branquinho
Andreana, Peter R.
Andreasen, Peter A.
Andrés, Maria Fe
Andrisano, Vincenza
Angela, Bisio
Anibal, Paula
Anissimov, Yuri G.
Anna, Horszwald
Annaert, Pieter
Anraku, Makoto
Antipenko, Lyudmila N.
Antje, Huefner
Antonelli, Alessandro
Anzai, Jun-ichi
Apone, Fabio
Aponick, Aaron
Arai, Masayoshi
Arano, Yasushi
Archibald, Steve J.
Arct, Jacek
Arellano, Juan B.
Arenas, Miguel
Ariga, Katsuhiko
Arosio, Daniela
Arratia-Pérez, Ramiro
Arrieta, Jesús
Aruoma, Okezie
Asai, Akira
Asai, Teigo
Asea, Alexzander
Asís, Silvia E.
Assimopoulou, Andreana N.
Astakhova, I. Kira
Asti, Mattia
Ata, Athar
Atrian, Sílvia
Attioua, Barthelemy K.
Aullon, Gabriel
Avarvari, Narcis
Avato, Pinarosa
Avula, Bharathi
Ayala-Zavala, J. F.
Aydemir, Adnan
Ayyalusamy, Ramamoorthy
Azam, Faizul
Ba, Yong
Baba, Akio
Babula, Petr
Bach, Horacio
Bachran, Christopher
Baczek, Tomasz
Baek, Hyung Hee
Baek, Nam In
Baeza, Alejandro
Bagnoli, Luana
Bahn, Andrew
Bai, Hanying
Bai, Meng-Yi
Baiano, A.
Baker, C. Jacyn
Baker, Michael E.
Bal, Wojciech
Balagiannis, Dimitrios
Baldwin, Elizabeth
Balemba, Onesmo B.
Ballatore, Carlo
Balling Engelsen, Søren
Baltina, L. A.
Baltora-Rosset, Sylvie
Balzarini, Jan
Bando, Yasuko
Banerjee, Sanjeev
Bang, Jeong Kyu
Banghart, Matthew
Bánóczi, Zoltán
Bansal, Ranju
Banskota, Arjun H.
Bao, Chongyun
Bao, Ming
Bao, Yongping
Baqi, Younis
Barbas, Carlos F.
Barbera, Mariagnese
Barbosa-Pereira, Letricia
Barboza, M.
Bardi, Giuseppe
Bardón, Alicia
Barker, David
Barluenga, José
Barone, Giampaolo
Barr, John
Barreira, João
Barreto Silva, Fátima Regina Mena
Barreto-Bergter, Eliana
Barreto, Maria Do Carmo
Barrio, Pablo
Bass, Chris
Bastos, J. K.
Batanero, Belen
Batista, Ronan
Batovska, Daniela
Battino, Maurizio
Baudequin, Christine
Bavaresco, Luigi
Bavetsias, Vassilios
Baxendale, Ian
Bayse, Craig A.
Beaucage, Serge
Bechthold, Andreas
Beck-Sickinger, Annette
Beck, Melinda A.
Bednarczyk-Cwynar, Barbara
Bekhit, Aladin
Bell, Stephen
Bellinger, Rick
Belmonte, Mark
Ben-Shabat, Shimon
Bencini, Andrea
Bencivenni, Giorgio
Benites, Julio
Benke, Dietmar
Benkendorff, Kirsten
Bennet, Andrew
Benoist, Eric
Berestetskii, Berestetskii
Bergamini, Paola
Berger, Walter
Berlin, Darrell
Bermúdez-Aguirre, Daniela
Bermúdez-Torres, Kalina
Bernardi, Luca
Bernstein, Elliot R.
Bertelli, Davide
Berti, Debora
Bertolini, Joseph
Besson, Thierry
Best, Michael D.
Beutler, John A.
Bhat, Shridhar
Bhowmik, Prasanta
Bhushan, Alok
Bianchi, Antonio
Bielawski, Christopher W.
Biersack, Bernhard
Biesaga, Magdalena
Bijak, Michał
Bilsland, Elizabeth
Biot, Christophe
Birner-Gruenberger, Ruth
Biroccio, Annamaria
Bishayee, Anupam
Blaazer, Antoni R.
Blackburn, Michael
Blackstone, Neil W.
Blackwell, Helen E.
Blair, Richard G.
Blais, Anne
Blanot, Didier
Blažević, I.
Blindauer, Claudia A.
Boa, Andrew N.
Bobe, Gerd
Boersma, Hendrikus H.
Böhm, Volker
Bokoch, Michael P.
Bommareddy, Ajay
Bonifácio, Vasco
Boo, Yong Chool
Bordiga, Matteo
Bordoni, Alessandra
Borges, Rosivaldo
Boros, Laszlo
Borovkov, Nicholas Yu
Borrelli, Francesca
Borriello, Carmela
Botta, Mauro
Bouaziz, Serge
Bouet, Gilles
Boulanger, É.
Boulogne, Isabelle
Bouquillon, Sandrine
Bovell-Benjamin, A. C.
Brackman, Gilles
Bradshaw, Claire E.
Braissant, O.
Brandão, Maria das Graças
Brannon, Patsy M.
Braslavsky, Silvia E.
Brattström, A.
Braun, Klaus
Bravo, Héctor R.
Brazel, Christopher S.
Brazier, John A.
Breckenridge, Ross
Brecker, Lothar
Brehm, John
Breitling, Frank
Bremner, John B.
Brinker, Thomas
Broderick, Patricia A.
Brown, Andrew
Brown, Dan
Brown, Lindsay
Brown, Richard C. D.
Brudzynski, Katrina
Bruheim, Skjalg
Brüning, Ansgar
Brunner, Eike
Bruno, Maurizio
Brus, Jiří
Bruschi, Marcos
Brüschweiler, Rafael
Brusotti, G.
Brust, Peter
Bruzik, Karol
Bryant-Friedrich, Amanda
Bryant, Joseph
Bryce, David L.
Brycki, Bogumił
Brzezinski, Krzysztof
Bu, Xianhe
Bu, Xianzhang
Buchbauer, Gerhard
Büchel, Claudia
Budzisz, Elzbieta
Bühler, Bruno
Bujoli, Bruno
Bukowska, Bożena
Bundle, David R.
Buolamwini, John K.
Burger, Alain
Burghardt, Nesha S.
Burketova, L
Burks, Charles S.
Burt, Sa
Bustamante, J.
Bustos, Daniel A.
Buszek, Keith R.
Butin, Alexander V.
Butler, Alison
Byk, Gerardo
Caballero, Julio
Caboni, Pierluigi
Cabrera, Carmen
Cabrera, Margarita Gutiérrez
Čačić, Milan
Cadierno, Victorio
Cadwallader, Keith
Cai, Jianfeng
Cai, Lisheng
Cai, Shaoqing
Calderon, Angela
Caliandro, Rocco
Calín-Sánchez, Ángel
Calmès, Monique
Camacho-Corona, M. R.
Camara, Bilal
Câmara, José
Camargo, Carlos
Camins, Antoni
Campidelli, Stéphane
Campiglia, Pietro
Campos, Carmen A.
Campos, Joaquín M.
Canac, Yves
Candeias, Nuno R.
Canevotti, Renato
Canini, Antonella
Cantorna, Margherita
Cao, Jay J.
Cao, Song
Cao, Yanping
Caporale, A.
Cappelli, Chiara
Caputo, Gregory A.
Caraglia, Michele
Caramella, Carla
Cardile, V.
Cardoso, Susana M.
Carloni, Paolo
Carmona, Daniel
Carradori, Simone
Carrano, Carl J.
Carrasco-Pozo, Catalina
Carrillo, María Cristina
Carroll, Camille B
Carta, Antonio
Caruso, Francesco
Carvajal-Millan, Elizabeth
Carvalho, Ivone
Casas, L.
Casemiro, Luciana Assirati
Caskey, Charles F.
Castagné, Vincent
Castellano, José María
Castellari, Massimo
Castellarin, Simone D.
Castro, Angeles
Castro, Bruno M.
Castro, Helena Carla
Castro, Solange
Casu, M. Benedetta
Cavar, Sanja
Cavazza, Antonella
Cavicchi, Kevin
Cawkwell, Marc
Celzard, Alain
Cerecetto, Hugo
Ceron, Jose
Chabert, Philippe
Chan, Chin-Feng
Chan, Shun-Wan
Chan, Stephen L.
Chan, Thomas Y. K.
Chang, Cicero L. T.
Chang, Fang-Rong
Chang, Hsueh-Wei
Chang, Hyeun Wook
Chang, Il-Moo
Chang, Raymond
Chang, Sandra P.
Chang, Sue Joan
Chang, Tom
Chang, Young-Tae
Chang, Yuan Shiun
Chantrapromma, Suchada
Chapman, Eli
Chaves, Mariana
Chen, Baohua
Chen, Calvin Yu-Chian
Chen, Chiachung
Chen, Chien-Chih
Chen, Chung-Yi
Chen, D.
Chen, Fadi
Chen, Haifeng
Chen, Hubiao
Chen, Hui-Sheng
Chen, Ih-Sheng
Chen, Jhy-Der
Chen, Ji Jun
Chen, Jiangshan
Chen, Jianhan
Chen, Jianjun
Chen, Jih-Jung
Chen, Kai
Chen, Menglin
Chen, Nai-Hong
Chen, Oliver
Chen, Peng
Chen, Pin-Shern
Chen, Qi
Chen, Ruey-Shyang
Chen, S.
Chen, Wei-Min
Chen, Wen-Bin
Chen, Xi
Chen, Xuanmao
Chen, Yen-Ling
Chen, Ying
Chen, Yue
Chen, Zhe-Sheng
Cheng, Dandan
Cheng, Hsueh-Ling
Cheng, Kuan-Chen
Cheng, Ming-Jen
Cheng, Tak
Cheng, Tiejun
Cheng, Wei-Chieh
Cheng, Xin-Lu
Cheng, Yiyun
Cheng, Yongxian
Cheong, JaeHun
Chang. Chi-I.
Chi, Robin Yonggui
Chia, Brian
Chiang, Chiao-Hsi
Chiang, Hung-Lung
Chiang, John
Chiba, Kazuhiro
Chibiryaev, A. M.
Chien-Chih, Chiu
Chien, Yi-Chi
Chiou, Bor-Sen
Chiou, Shih-Hwa
Chiou, Wen-Fei
Chirea, Mariana
Chiti, Arturo
Chmielewski, Marek
Cho, Chan Sik
Cho, Yong Seo
Cho, Young Sik
Choi, Dong-Kug
Choi, Hyung-Kyoon
Choi, Jae-Hong
Choi, Jong Hyun
Choi, Kyoungju
Choi, Seok Ki
Choi, Yung Hyun
Choma, Adam
Chong, Youhoon
Chootip, Krongkarn
Choshi, Tominari
Chou, Duen-Suey
Choy, Ying
Choyke, Peter L.
Christensen, Søren Brygger
Chrostowska, Anna
Chu, Fang-hua
Chu, Justin
Chua, Lee Suan
Chuang, John Shih-ching
Chuman, Hiroshi
Chun, Ock
Cicco, Nunzia
Cielecka-Piontek, Judyta
Cimanga, R. K.
Cioli, Donato
Cirillo, Giuseppe
Clarés-Naveros, Beatriz
Clark, Daniel A.
Clarke, Anthony
Clarke, Donald
Cocco, Melanie
Cock, Ian E.
Cody, Jeremy
Coelho, Jaime
Coiffard, Laurence J. M.
Colca, Jerry R.
Coleman, Anthony W.
Coll, Josep
Collins, Shawn K.
Colom, Helena
Colombo, Maria Laura
Combes, Sébastien
Combrinck, S.
Comes-Franchini, Mauro
Conesa, J. A.
Conrad, Marcus
Consoli, Grazia M. L.
Contestabile, Antonio
Coppola, Thierry
Coquerel, Yoann
Corazza, Marcos L.
Corrêa, Arlene
Correia, Jack
Cosconati, Sandro
Cosgarea, Rodica
Cossignani, Lina
Cossio, Fernando
Costa, Susana P. G.
Costoya, Jose A.
Couce, María D.
Couture, Olivier
Couvineau, Alain
Cox, Liam R.
Creamer, Rebecca
Cresteil, Thierry
Crews, Colin
Crich, Simonetta Geninatti
Cristofari, Gael
Crowley, James D.
Crupi, P.
Cruz, Jader S.
Csuk, René
Cuca, Luis E.
Cuerva, Juan M.
Culmsee, Carsten
Cultrone, Antonietta
Cumpstey, Ian
Cunha, Anna C.
Cunico, Wilson
Curcio, Manuela
Custodio, Luisa
Cuzaocrea, Salvatore
Czech-Kowalska, Justyna
D'abrosca, Brigida
D'ambrosio, Michele
D'Andrea, Luca D.
Dahlberg, Clinton J.
Dai, Zong
Dall´Acqua, Stefano
Dallinger, Doris
Damatta, Fabio M.
Daming, Du
Daniele, Simoni
Daniels, Matthew H.
Danishefsky, Samuel J.
Danova, Kalina
Dantas, Renato F.
Dante, Roberto C.
Dauncey, M.
Davies, Stephen G.
De Almeida Barbosa, L. Claudio
De Andrade e Silva, Suheyla
De Beer, Dalene
De Beer, J.
De Carvalho, Carla
De Feo, vincenzo
De Freitas, Rivelilson Mendes
De Groot, Herbert
De Kimpe, Norbert
De La Cruz, V. Pérez
De La Fuente, M. A.
De La Hoz, Antonio
De Lago, Eva
De Luca, Vincenzo
De Luna Martins, Daniela
De Mendonça, Dina I. M. D.
De Moliner, Fabio
De Oliveira Monteiro, Caio Márcio
De Oliveira, Helena C. F.
De Oliveira, Luiz Fernando C
De Paz, Jose Luis
De Quirós, A. Rodríguez-Bernaldo
De Robertis, Riccardo
De Sousa, Orlando Vieira
De Souza, Ana Olívia
De Souza, Gezimar D.
De Spiegeleer, Bart
De Villiers, André
De Vrese, Michael
De With, Gijsbertus
Debbert, Stefan L.
Debnath, Samir
Décaudin, Bertrand
Defrancq, Eric
Dehaen, Wim
Delgado, Francisco J.
DellaGreca, Marina
Delogu, Lucia Gemma
Delsart, Cristèle
Dembinski, Roman
Demopoulos, Vassilis J.
Denny, Bill
Denti, Michela A.
Desaulniers, Jean-Paul
Desbois, Nicolas
Desmaële, Didier
Desreux, Jean F.
Destefano, J. J.
Detrembleur, Christophe
Devesa, V.
Devi, M. Geetha
Devreese, Mathias
Dhawan, Deepika
Di, Rong
Diamond, Gill
Diana, Patrizia
Dias, Bernardo
Diaz-Lanza, Ana Maria
Diaz, Isabel
Díaz, Mario
Diez, David
Dijkstra, Pieter J.
Dijkwel, Paul
Dilecce, Giuseppe
Dilis, Vardis
Ding, Gang
Ding, Jian
Ding, Jinsong
Ding, Ke
Ding, Xiaochu
Dinglasan, Rhoel R.
Dixit, Bharat C.
Djarova, Tryana
Dodziuk, Helena
Doligalska, M.
Domingo, Luis R.
Dominici, Paola
Doña-Rodríguez, José M.
Dondoni, Alessandro
Dong, Yuesheng
Donner, Christopher D.
Donno, D.
Donsì, Francesco
Donzello, Maria Pia
Dooley, S.
Dragutan, Ileana
Dragutan, Valerian
Drosatos, Konstantinos
Du, Rose
Du, Yuguo
Du, Yunfei
Duan, Jinao
Duarte, Whasley Ferreira
Düchler, Markus
Dueňas-González, A.
Duerkop, Axel
Duh, Pin-Der
Dumoulin, F.
Duncan, Robin E.
Duodu, Kwaku G.
Durán-Patrón, Rosa
Dwivedi, Chandradhar
Dykes, Gary A.
Easmon, Johnny
Ebel, Rainer
Ebinger, Katalin
Ebrahimi, Azadeh
Echevarria, Aurea
Eckert, Heiner
Eckl, Peter M.
Eichen, Yoav
Eichler, Jutta
Eifler-Lima, Eifler-lima
Eitsuka, Takahiro
Eklund, Patrik C.
Eklund, Per
El Kaïm, Laurent
El-Achkar, Tarek
El-Amri, Chahrazade
El-Readi, Mahmoud Zaki
El-Seedi, Hesham R.
Eller, Fred
Elmegeed, Gamal A.
Elofsson, Mikael
Elwakeel, Khalid Z.
Engels, Joachim W.
Enjoji, Munechika
Enríquez, Raúl G.
Erdélyi, Máté
Ermert, Johannes
Escalante, Jaime
Escolano, Carmen
Escriche, Isabel
Escuredo, Olga
Esnault, Vincent L. M.
Espinosa-Aguirre, Esús Javier
Esquivel, Rodolfo O.
Ess, Daniel H.
Estevinho, Leticia
Etzkorn, Manuel
Evans, Amanda C.
Faber, Kurt
Fahmy, Hesham T. Y.
Faik, Ahmed
Fallarero, Adyary
Fan, Enguo
Fan, Mingtao
Fan, Timothy M.
Fan, Yunchang
Fan, Zhijin
Fanning, Kent
Farid, Chemat
Fariña, Julia I.
Farr, Susan A.
Fasinu, Pius S.
Fausto, Rui
Favi, Gianfranco
Fayet, G.
Feás, Xesús
Felluga, Fulvia
Felsenstein, Kevin M.
Feng, Meiqing
Feng, Yangbo
Feng, Yibin
Feo, Francesco
Feresin, Gabriela E.
Feringa, Ben L.
Fernand, Vivian
Fernandes, K. F.
Fernandez Megia, Eduardo
Fernández-Alba, A. R.
Fernández-Arroyo, Salvador
Fernandez-Lafuente, Roberto
Fernández-Miyakawa, M. E.
Fernandez-Recio, Juan
Fernández, José J.
Fernández, Rosario
Feron, Gilles
Ferrari, Stefania
Ferreira, Juliana
Ferreira, Marcelo J. P.
Ferreira, Susana
Fiala, André
Fickers, Patrick
Figadere, Bruno
Figueiredo, Ana Cristina
Filarowski, Aleksander
Filippov, Dmitri V.
Fiorucci, Stefano
Fischer, Reinhard
Flaherty, Patrick T.
Fliegner, Daniela
Florea, Bogdan I.
Florence, Val
Fondjo, Emmanuel Sopbué
Font, A.
Font, María
Fontana, Antonella
Fontes, Ana
Forano, Evelyne
Fornari, Francesca
Fornari, Tiziana
Fortunatia, E.
Foster, J. A.
Foti, Robert S.
Foubelo, Francisco
Fragai, Marco
Francia, Silvia De
Francis, Heather
Francis, I. Leigh
Franco, Octávio Luiz
Frappier, Lori
Frau, Juan
Freile, Mónica L.
Freitas, Ana Cristina
Fristrup, Peter
Froldi, Guglielmina
Frolov, Andrej
Frontera, Antonio
Frontier, Alison J.
Fryxell, Glen
Fu, Hua
Fu, Li-Wu
Fu, Shaohai
Fu, Tzu-Fun
Fu, Zhifeng
Fuguet, Elisabet
Fujii, Katsuhiko
Fujimoto, Yoshinori
Fujita, Masaki J.
Fujita, Yoshio
Fukuda, Michiko
Fukusaki, Eiichiro
Fukuyama, Tomoki
Fulgosi, Hrvoje
Furuzono, Tsutomu
Fuselli, Fabio
Fusi, Vieri
Fuwa, Haruhiko
Gabriele, Bartolo
Galagudza, Michael
Galán-Vidal, Carlos
Galat, A.
Galati, Enza Maria
Galderisi, Umberto
Gali-Muhtasib, Hala U.
Gallagher, John F.
Galvão, Adelino M.
Gambino, Dinorah
Gamcsik, Michael P.
Gamel, Tamer
Gan, Liangbing
Gandhi, Neha
Gannett, Peter
Gao, Yanxiang
Gao, Feng
Gao, Haixiang
Gao, Jin-Ming
Gao, Kun
Gao, Min
Gao, Shuanhu
Gao, Wen-Yuan
Gao, Xiumei
Gao, Yuan
Garaj, Vladimír
GarcÃ­a-Domenech, Ramon
García-Álvarez, Joaquín
García-Martinez, Joaquín C.
García-Parrilla, M. C.
García-Valverde, María
García-Villanova, Belén
Garcia, R.
Garozzo, Adriana
Garraud, Olivier
Garre, Dulcenombre Gómez
Garrote, Gil
Gáspári, Zoltán
Gaunitz, Frank
Gauthier, Charles
Gawlik-Dziki, Urszula
Gawrisch, Klaus
Gbogouri, Albarin
Geierstanger, Bernhard H.
Gein, V. L.
Gelmi, Maria Luisa
Gendaszewska-Darmach, Edyta
Geneviève, Bourdy
Gennäs, Gustav Boije Af
Georgiev, Milen
Gerbaux, Pascal
Geronikaki, Athina
Gerós, Hernâni
Gerwick, Lena
Geso, Moshi
Gestwicki, Jason E.
Ghandi, Khashayar
Ghilardi Lago, João Henrique
Ghosh, Dipankar
Giancola, Concetta
Gibbs, Roz
Giernoth, Ralf
Gikas, E.
Gil, Jose´ I.
Gilani, Anwar
Gilmer, John F.
Gimalova, F. A.
Giménez, Dolores García
Giménez, Julieta Pérez
Giménez, María S.
Gimpl, Gerald
Gioiello, Antimo
Giralt, Ernest
Girbés, Tomás
Gising, Johan
Giuffrida, Daniele
Giunchedi, Paolo
Glaser, Keith B.
Glauser, Gaetan
Gledhill, Martha
Gobis, Katarzyna
Godt, Adelheid
Gomes, Valdirene Moreira
Gómez-Caravaca, Ana María
Gómez-Gómez, Lourdes
Gonçalves, M. Sameiro T.
Gong, Xingchu
Gong, Young-Dae
González-Aguilar, Gustavo Adolfo
González-Gómez, D.
González-Marrero, Joaquin
González, Concepción C.
González, María Pilar
Goping, Ing Swie
Gordaliza, M.
Gordon, Michael
Gorjanović, Stanislava Ž.
Gorman, Gregory S.
Gorzalczany, S.
Goslinski, Tomasz
Gosmann, Grace
Gossage, E. R. T.
Goti, Andrea
Gotor-Fernández, Vicente
Gottesman, Michael M.
Gottfried, Carmem
Gou, Shao-Hua
Gourier, Didier
Gourley, Shannon L.
Goya, Luis
Grabchev, Ivo
Graber, David J.
Graether, Steffen P.
Graf, Robert
Grandas, Anna
Granet, Robert
Granica, Sebastian
Grant, Stephan
Grassi, Alfonso
Grassi, Mario
Gratteri, Paola
Gratzl, Miklos
Graves, David
Green, Ezekiel
Greenberg, Robert M.
Greenberger, Joel S.
Greer, Charles August
Greisch , Jean-Francois
Grenier, Nicolas
Grieser, Franz
Griffith, Renate
Griffiths, William
Grimaldi, Maurizio
Grinberg, Sarina
Groppo, Francisco Carlos
Gros, Claude P.
Gruber, Christian
Grune, Tilman
Gruselle, Michel
Grzebisz, Witold
Gu, Yong
Gu, Zhongwei
Gude, Veera Gnaneswar
Gudiminchi, Rama
Guénin, Erwann
Guerrero, Sergio A.
Guidi, Lucia
Guil-Guerrero, Jose
Guilhon, Giselle Maria S. P.
Guillaume, Dominique
Guillemette, T.
Guillemin, Jean-Claude
Gülçin, İlhami
Gunstone, Frank D.
Guo, Jia
Guo, Ju-Tao
Guo, Qiaosheng
Gurvich, Vadim J.
Gutiérrez-Uribe, Janet A.
Gutierrez, Rosa Martha Perez
Guzmán, Esther A.
Hackenberg, Michael
Hada, Kihito
Hadad, Mohamed
Hadjipavlou-Litina, Dimitra
Haertlé, Thomas
Haider, Norbert
Haiges, Ralf
Haino, Takeharu
Hak, Sjoerd
Hakamata, Hideki
Halasz, Ivan
Halford, Nigel G.
Hall, Marilena
Hallouard, François
Hamana, Hiroshi
Hamid, N.
Hamme Ii, Ashton T.
Hampel, Daniela
Han, Jaehong
Han, Jin
Han, Quan-Bin
Han, Sang-Bae
Han, Weidong
Handa, Avtar K.
Hanessian, Stephen
Hanhineva, Kati
Hano, Christophe
Hanrahan, Grady
Hansen, D. Flemming
Hansen, Michael Ryan
Hanson, Susan K.
Hao, Da-Cheng
Harding, Wayne W.
Harper, Jason B.
Harris, Philip
Harrison, William
Hartley, C. Scott
Hartmann, Martin
Hasegawa, Tomonobu
Hashimoto, Masaru
Hassan, Mohamed
Hatae, Noriyuki
Hatakeyama, Susumi
Hatano, Tsutomu
Havet, Jean-Louis
Hayashi, Yoshihiko
Haybaeck, Johannes
He, Hong-Ping
He, Langchong
He, Liangnian
He, Xiao-Peng
He, Yanhong
Hearn, Michael J.
Heasley, Brian H.
Hedges, Jodi
Hegedűs, A.
Heilig, Andrej
Henary, Maged
Henderson, Luke
Henryk, Kozlowski
Heras, Ángeles
Herczegh, Pál
Herdewyn, Piet
Herling, Marco
Hermosa, Rosa
Herrador, M. Mar
Herraiz, T.
Heuberger, Eva
Heuzey, Marie-Claude
Hider, Robert C.
Higman, Vicky
Hijji, Yousef
Hindsgaul, Ole
Hipkiss, Alan R.
Hirano, Tomoya
Hirao, Akira
Hirayama, Masao
Hiroaki, Hidekazu
Hiruma-Lima, Clelia
Ho, Chi-Tang
Ho, Feng-Ming
Ho, Mei-Lin
Ho, Su-Chen
Höbartner, Claudia
Hoffman, Sebastien
Hofmann, Alan F.
Hogg, Philip
Hohlfeld, Thomas
Hojo, Hironobu
Højrup, Peter
Hollung, K.
Holmgren, Arne
Holzgrabe, Ulrike
Honda, Hisashi
Honda, Tadashi
Hondal, Robert J.
Hong, Jiann-ruey
Hong, Jongki
Hong, Liangzhi
Hong, Mei
Hong, Yonggeun
Honker, Hans
Horng, Jim-Tong
Horobin, Richard W.
Hort, Mariana Appel
Hoshino, Tyuji
Hosni, Karim
Hossain, Mohammad B.
Hou, Chien-Wen
Hou, Wen-Chi
Howard, Zack
Howell, Amy R.
Howes, Melanie
Hozák, Pavel
Hrckova, Gabriela
Hromadkova, Z.
Hseu, You-Cheng
Hsieh, Hsing-Pang
Hsieh, Jen-Chien
Hsieh, Yi-Hsien
Hsing, C.-H.
Hsu, Day-Shin
Hu, Bing
Hu, Miao-Lin
Hu, Miao-Lin (Merlin)
Hu, Tianhui
Hu, Wei-Wen
Hu, Xiang-Ping
Hu, Xiaowen
Hu, Yonghong
Hu, Zhe-Yi
Hua, Daoben
Hua, Erbing
Hua, Huiming
Huang, Chang-Gan
Huang, Chi-Chang
Huang, Feihe
Huang, Jinhua
Huang, Mingdong
Huang, Xuefei
Huang, Ya-Ling
Huang, You
Huang, Zhen
Huang, Zhibin
Huber, Thomas
Huczyński, Adam
Hudson, Bruce
Hui, Kam M.
Hulme, Christopher
Hunger, Johannes
Hunyadi, Attila
Huq, Fazlul
Hurvois, Jean-Pierre
Hussain, A.
Hwang, Baik
Hwang, Kyung A.
Hwu, Jih Ru
Hyafil, Fabien
Hyun, Myung Ho
Ibrahim, S. A.
Ichihashi, Masamitsu
Ifa, Demian
Iglésias, Bernardo
Ikejima, Kenichi
Im, Sin-Hyeog
Imai, Shinjiro
Imbimbo, Bruno P.
Immenschuh, Stephan
Inamoto, Kiyofumi
Inoue, Koichi
Iorizzi, Maria
Iqbal, Zafar
Isaksson, Dan
Isasi Allica, José Ramón
Ishibashi, Masami
Ishihara, Kazuaki
Ishikawa, Tomoyasu
Ishikura, Minoru
Ishimoto, Takayoshi
Ishman, P. F
Islam, Shahidul
Ismaiel, Omnia
Isman, M. B.
Isshiki, Yoshiaki
Ito, Hiroyasu
Ito, Takeo
Itoh, Ken-Ichi
Ittner, Lars M.
Ivanov, Ivo C.
Ivanovska, Nina
Iwabuchi, Yoshiharu
Iwagawa, Tetsuo
Iwamori, Masao
Iwashina, Tsukasa
Izquierdo, Luis
Jaakola, Laura
Jacqz-Aigrain, Evelyne
Jafarpour, Farnaz
Jagerovic, Nadine
Jagodziński, Paweł P.
Jampilek, Josef
Jänis, Janne
Jarosz, Slawomir
Jäschke, Andres
Jaworska, Malgorzata
Jean De Dieu, Tamokou
jean, Ludovic
Jeandet, Philippe
Jeng, Ru-Jong
Jensen, Søren Rosendal
Jeon, Jae-Gyu
Jeon, You-Jin
Jeong, Hyun-Ja
Jessen, Henning Jacob
Jezek, Jan
Ji, Shun-Jun
Jia, Cheng-Sheng
Jia, Xinru
Jiang, Hongliang
Jiang, Lin
Jiang, Xuchuan
Jiang, Yuyang
Jianhui, Qiu
Jiao, Guangling
Jim Simpson, Jim Simpson
Jimbo, Mitsuru
Jiménez-Barbero, Jesús
Jiménez, Jara Pérez
Jimeno, Ciril
Jin, Lijian
Jin, Weijun
Joe, Young Ae
Johansen, Kenneth T.
Johnson, Jeremy J.
Johnson, John A.
Jones, Christopher E.
Jonnalagadda, Subash C.
Joo, Myungsoo
Jordan, Joaquin
Jordão, António M.
Jóźwiak, Krzysztof
Juillerat-Jeanneret, Lucienne
Jullian, Valérie
Jung, Jiyoung
Jung, Youngmi
Junkers, Thomas
Juszczak, Lesław
Kachlicki, Piotr
Kai, Guoyin
Kajihara, Yasuhiro
Kakkar, Ashok K.
Kaliora, Andriana C.
Kalogeropoulos, Nick
Kalvins, Ivars
Kamel, Alaa
Kami, Daisuke
Kamio, Michiya
Kammerer, Robert
Kamolz, Lars P.
Kanakis, George
Kang, ChulHee
Kang, Congbao
Kang, Dawon
Kang, Jonghoon
Kang, Sam Sik
Kapoor, Mahendra P.
Karagiannis, Tom C.
Karvembu, R.
Kashiwada, Yoshiki
Kästner, Johannes
Kataoka, Takao
Kataoka, Yosky
Katarg, Argyropoulou
Katayama, Hidekazu
Kato, Eisuke
Kato, Masako
Katuchova, Jana
Kaurinovic, Biljana
Kawabata, Saneyuki
Kawabe, Takefumi
Kawanishi, Noriaki
Kay, Brian K.
Kazakova, O. B.
Keller, Thomas H.
Kendrick, John
Kerkhoff, Claus
Kerr, Phil
Kerwin, Sean M.
Keul, Helmut
Keyzers, Rob
Khalaf, Abedawn I.
Khalifa, Mohamed E.
Khan, Khalid Mohammed
Khan, N. A.
Kharwar, Ravindra
Khasawneh, Mohammad
Kikuzaki, Hiroe
Kim, Bumseok
Kim, Dong-Myung
Kim, Guntae
Kim, Hyung Min
Kim, Jin
Kim, Jin Moon
Kim, Jin Sook
Kim, Jung-Ae
Kim, Junheon
Kim, Minsoo
Kim, Nacksung
Kim, Soo-Un
Kim, Sung Yeon
Kim, Sung-Hoon
Kim, Uh-Hyun
Kim, Y. D.
Kim, Yeong Shik
Kim, Youngmee
Kimata-Ariga, Yoko
Kimura, Hideto
Kingsbury, Joanne M.
Kirby, Anthony
Kirby, Stephen D.
Kirsch, Gilbert
Kiso, Makoto
Kiso, Yoshiaki
Kiss, Anna K.
Kitamura, Tsugio
Kiuru, Paula
Kjaer, Anders
Klajnert, B.
Klapper, M.
Klein, Christian D.
Kleinpeter, Erich
Klinman, Judith P.
Klochkov, S. G.
Kloucek, Pavel
Kluza, J.
Knapp, Spencer
Knauer, Shirley K.
Knölker, Hans-Joachim
Knoshaug, Eric
Knudsen, Lotte Bjerre
Kobayashi Japan, Hidekazu
Kobayashi, Keiji
Kobayashi, Yuichi
Kodama, Hiroyuki
Kohaar, Indu
Kohen, Amnon
Kojima, Akiko
Kojima, Chie
Kojima, Chojiro
Kojo, Shosuke
Kolaczkowski, Marcin
Kolbe, L.
Kolehmainen, Erkki
Kolodziejczyk, Joanna
Komar, Anton A.
Kong, De-Ming
Kong, Dejuan
Kong, Ling-Yi
Konrath, Eduardo Luis
Koóš, Miroslav
Kopka, Klaus
Kopp, B.
Koppel, Kadri
Košak, Aljoša
Koschella, Andreas
Kosterink, Jos G. W.
Kostikov, Alexey P.
Kostogrys, Renata
Kotani, Shunsuke
Koyama, Tomoyuki
Kozikowski, Alan P.
Kozliak, Evguenii I.
Kozlov, Igor A.
Kozlowski, Henryk
Krajcsi, Peter
Krämer, Oliver H.
Kredics, Laszlo
Kremer, Dario
Krstic, Natalija
Kruger, Marlena
Kuang, Shihuan
Kubíček, Vojtěch
Kubo, Takuya
Kubo, Yuji
Kubota, Yasuhiro
Kucharikova, Sona
Küçükbay, Hasan
Kühn, Fritz E.
Kühn, Oliver
Kukovec, Boris-Marko
Kulik, Eva M.
Kulisic-Bilusic, Tea
Kulozik, Ulrich
Kumar, Manoj
Kumar, Rakesh
Kumar, Sanjai
Kumar, Satish
Kung, Hank F.
Kuniyasu, Akihiko
Kuo, Ping-Chung
Kuo, Sheng-Chu
Kuo, Yao-Haur
Kupcewicz, Bogumiła
Kurek, Mia
Kuroda, Minpei
Kurtán, Tibor
Kurtboke, Ipek
Kushida, Hirotaka
Kushiro, Tetsuo
Kvansakul, Marc
Kwon, Il-Keun
Kwon, Jung-Hwan
Kwon, Young-In
Kwon, Youngjoo
Kwong, Fuk Yee
La Clair, James
La Cruz, Thomas E.
Lacaille-Dubois, Marie-Aleth
Lafont, René
Lai, Chih-Ho
Lai, Daowan
Lai, Hung-Cheng
Lai, Jui-Yang
Lam, Jacky W. Y.
Lämmerhofer, Michael
Lammich, S.
Lang, Siegmund
Lange, Oliver
Langer, Oliver
Langer, Swen
Laplante, Mathieu
LaPlante, Steven R.
Larionov, Oleg V.
Larsen, Scott D.
Lasunción, Miguel A.
Laufer, Stefan
Lauria, Antonino
Lavine, Barry
LaVoie, Edmond J.
Lawson, Charlotte
Lazar, Diana
Lazar, S.
Lazareva, Nataliya F.
Lazaridis, Themis
Lazzarato, Loretta
Leardkamolkarn, Vijittra
Lebovka, N. I.
Lee, Dong Gun
Lee, Francis Young-In
Lee, Hangu
Lee, Hsinyu
Lee, Hyi-Seung
Lee, Jae Yeaol
Lee, Jae-Ho
Lee, Jaehwi
Lee, Jeong-Chae
Lee, Jin-Ching
Lee, Jin-Woo
Lee, Jiseok
Lee, Kyung-Yeol
Lee, Mi Kyeong
Lee, Min
Lee, Moses
Lee, Myung G.
Lee, Ok-Hwan
Lee, Rebecca
Lee, Ren-Shen
Lee, Seokjoon
Lee, Shim Sung
Lee, Soo Young
Lee, Zang Hee
Lee, Zhi-Hong
Legraverend, Michel
Lehmler, Hans-Joachim
Lehocky, Marian
Lei, Pingsheng
Lei, W.
Lelonek, Malgorzata
Len, Christophe
Lenardão, Eder J.
Lentini, Giovanni
Lentjes, E. G. W. M.
León-Rivera, Ismael
Leonard, C. M.
Leonelli, Francesca
Leonetti, Francesco
Leong, Lai Peng
Leonidas, Demetres D.
Lépine, Francis
Lertsiri, Sittiwat
Lespade, Laure
Lesyk, Roman
Leu, Yann-Lii
Leung, Chung-Hang
Leung, Dennis H.
Leung, Euphemia
Leung, Kar W.
Leung, Pc
Lewinska, Anna
Lewis, Gareth
Li, Cheng
Li, Chien-Ming
Li, Chuan
Li, Chuangchuang
Li, Dehai
Li, Fuyou
Li, George Qian
Li, Huabin
Li, Huaming
Li, Huihui
Li, Jian
Li, Jianrong
Li, Jianshu
Li, Jing
Li, Kecheng
Li, Lin
Li, M. X.
Li, Meng-Hui
Li, Nianguang
Li, Ning
Li, Peiwu
Li, Pengcheng
Li, Rui
Li, Shao-Shun
Li, Shenshen
Li, Wei
Li, Xiaoyu
Li, Yanfeng
Li, Yaping
Li, Yi
Li, Yiming
Li, Yingwei
Li, Yuliang
Li, Zibo
Liang, Po-Huang
Liang, Tingbo
Liang, Xin-Miao
Lião, Luciano M.
Liao, Wuping
Liao, Yang
Liao, Yong-Hong
Liebscher, Juergen
Liekens, Sandra
Liese, Andreas
Liguori, Angelo
Lillich, James D.
Lim, Beong Ou
Lim, Yau-Yan
Lim, Yoongho
Lima, Lídia Moreira
Lin, Chih-Chien
Lin, Chun-Cheng
Lin, Chun-Ching
Lin, Chun-Yuan
Lin, Hai-Shu
Lin, Hsiang-Ru
Lin, Jingming
Lin, Kuo-I.
Lin, Nai-Chun
Lin, Shyh-Hsiang
Lin, Tzu-Chau
Lin, Wen-Han
Lin, Xufeng
Lin, Ying-Chih
Lin, Yun-Lian
Lin, Yun-Wei
Lin, Zhenjian
Linclau, Bruno
Lincoln, Per
Lindhorst, Thisbe
Lindoy, Leonard
Ling, Jian Ya
Ling, Li
Liou, Jing-Ping
Liou, Kuo-Tong
Liskamp, Rob M. J.
Listrat, Anne
Litinas, Konstantinos
Litonjua, Augusto A.
Litvić, Mladen
Litwinienk, Grzegorz
Liu, Fang
Liu, Hai-Yang
Liu, Ji-Bin
Liu, Jiang-Xun
Liu, Jie
Liu, Jingze
Liu, Lei
Liu, Liang
Liu, Pengzhan
Liu, Ruifeng
Liu, Sean X.
Liu, Shing-Hwa
Liu, Yonghong
Liu, Yongjian
Liu, Yongjun
Liu, Yung-Chuan
Liu, Yunling
Liu, Zai-Qun
Liu, Zhi Long
Liu, Zhi-Pei
Liu, Zhongfa
Lizard, Gérard
Lo Scalzo, Roberto
Lo, Jeng-Fan
Lobo-Castañón, Maria Jesus
Locatelli, Marcello
Lodovici, Maura
Longley, Db
Loo, Alvin
Lopes, Dinora
López, Carlos Silva
López, Carmen Remuñán
Lopez, Fernando
López, Luis A.
López, Víctor
Lorenz, Peter
Los, Marek
Löser, Reik
Lotina-Hennsen, Blas
Lovero, Grazia
Lowary, Todd L.
Lowe, Gordon
Lu, Chengping
Lu, Chung-Dar
Lu, Dah-Yuu
Lu, Jin-Jian
Lu, Jun
Lu, Long
Lu, Mangeng
Lu, Pei-Luen
Lu, Rong
Lu, Wei
Lu, Yong
Lu, Yunhua
Lu, Zhaoxin
Lucas, Pierre
Lucinda, Leda Marília Fonseca
Lüdtke, Diogo S.
Ludvík, Jiří
Lugo-Cervantes, Eugenia
Lui, Ho Chi
Luo, Qun-Li
Lupiáñez, José A.
Luque, Rafael
M. Nyström, Andreas
Ma, Chaomei
Ma, Cheng
Ma, Choong
Ma, Edmond Dik-Lung
Ma, Enlong
Ma, Guang-Hui
Ma, Sen
Ma, Shengqian
Macleod, Roderick A. F.
Maddocks, Sarah E.
Madej, Katarzyna
Maeda, Kiminori
Maeda, Toshinari
Maes, Louis
Maffei, Massimo
Maga, Giovanni
Magazu, Salvatore
Magedov, Igor V.
MaGee, David I.
Maggi, F.
Magid, Abdulmagid Alabdul
Mahana, Carlos David Pessoa
Mahatthananchai, Jessada
Maher III, L. James
Maher, Pamela
Maia, Cristiane Do Socorro Ferraz
Mak, Nai K.
Makala, Levi H. C.
Mäki-Arvela, Päivi
Mäkinen, Ville-Petteri
Makker, Pratima Nangia
Makriyannis, Alexandros
Maksimov, I. V.
Malchiodi, Emilio L.
Malekinejad, H.
Malheiro, Ricardo
Malo, C.
Malterud, Karl Egil
Mamat, Constantin
Mambu, Angèle Lengo
Mangalagiu, Ionel I.
Mani, Sridhar
Manna, Prasenjit
Manoury, Eric
Mantegazza, Francesco
Mantell, C.
Manzhos, Sergei
Mao, Jian-Hua
Mao, Jingdong
Mara, Freire
Maranzana, Andrea
Marchal, Juan A.
Marchan, Jonathan S.
Marchioni, Eric
Marco-Contelles, José
Margarity, Marigoula
Mariano-Oliveira, Andréa
Marik, Jan
Marin, M. Luisa
Marini-Bettolo, Rinaldo
Markopoulos, John
Markopoulou, Olga
Markwick, Phineus R. L.
Marnett, Larry J.
Marra, Alberto
Marrubini, Giorgio
Marsh, Ken
Marsolais, Frédéric
Marti, Sergio
Martín-Cordero, Carmen
Martín-Mazuelos, Estrella
Martin, Daniel
Martin, Rachel
Martin, Santiago
Martinelli, Adriano
Martínez-Velázquez, Moisés
Martinez, Ana
Martínez, Ana
Martínez, Ana M.
Martínez, Tomas
Martino, Sabata
Martins, Albino
Martins, Carlos
Maruyama, Toru
Marzaro, Giovanni
Masanori, Terasaki
Massiot, Georges
Masu, Hyuma
Mata, I.
Mata, Jose A.
Matan, Narumol
Materska, Małgorzata
Mathesius, Ulrike
Mathew, Thomas
Matich, Adam J.
Matlawska, Irena
Matos, Maria J.
Matosiuk, Dariusz
Matsuda, Hisashi
Matsuda, Yudai
Matsuo, Yosuke
Matsuura, Hideyuki
Matus, José Tomás
May, Brian
Mayr, Christine
Mazerska, Zofia
Mazutti, Marcio A.
Mazzeo, Mina
Mbeunkui, Flaubert
McAlpine, Shelli
McCarthy, J. M.
Mcdougall, Gordon J.
Mckallip, Robert J.
McKee, Tawnya C.
Mckenzie, Iain
Mclean, John H.
Medana, Claudio
Mehmetoğlu, Ülkü
Mehrvar, Mehrab
Meierjohann, Axel
Meijer, Bert
Meiler, Jens
Melagraki, Georgia
Mellor, Andrew L.
Menaa, Farid
Mencherini, Teresa
Menezes, José
Meng, Fan-Yi
Merino, Pedro
Mesías, Marta
Messeguer, Angel
Messori, Luigi
Meyer, Matthew P.
Meyer, R.
Miao, Lu
Miao, Zhiwei
Miceli, Antonio
Michaelakis, Antonios
Michele, Baglioni
Micura, Ronald
Miguel, M. G.
Mikkelsen, Deirdre
Mikulski, Damian
Milewski, Sławomir
Milhauser, Glenn
Miller, Stephen C.
Mimaki, Yoshihiro
Min, Byung Sun
Minoura, Mao
Minto, Robert E.
Miomandre, Fabien
Miquel, Pons
Miranda, L.
Mireles Dewitt, Christina A.
Misra, Devesh
Misra, Sougat
Mitsunaga, Tohru
Miura, Yutaka
Miyata, Okiko
Miyazaki, Kouji
Miyoshi, Daisuke
Mizuno, Masashi
Mladěnka, Přemysl
Młochowski, J.
Mlostoń, Grzegorz
Moberg, Christina
Moderhack, Dietrich
Modica, Maria
Moeslinger, Thomas
Mohallem, Nelcy Della Santina
Mohammad, Ramzi M.
Mohan, Ram S.
Mohareb, Raafat
Moine, Laurence
Moineau-Chane-Ching, Kathleen I.
Moliner, Vicent
Möller, Heiko
Molnár, Péter
Monchaud, David
Moncol, Jan
Monge, David
Monopoli, Antonio
Montange, Damien
Montaut, Sabine
Monte, Francisco
Monteiro, Luís S.
Montesarchio, Daniela
Montón, Helena
Montoro, Paola
Moodley, Brenda
Moon, Hyung Ryong
Moon, Young-Choon
Moorthy, N. S. Hari Narayana
Moosmann, B.
Morales, Melissa
Morán, Lara
Morel, Ademir F.
Morel, Nicole
Moreno, Silvia
Moretto, Alessandro
Morgado, Jorge
Mori, Seiji
Morikawa, Toshio
Morris, John Steven
Mort, John S.
Moshi, M. J.
Moss, Mark
Motoo, Yoshiharu
Motti, Cherie
Motz, Vicki Abrams
Mourtas, Spyros
Moussaoui, Y.
Moutsatsou, P.
Moyano, Albert
Mu, Linjing
Muddiman, David C.
Mueller-Harvey, Irene
Muhle, Claudia
Muise-Helmericks, Robin C.
Mulder, Patrick P. J.
Muller, C. J. F.
Muller, Christa
Müller, Thomas J. J.
Müller, Werner E. G
Murakami, Hiroto
Murakami, Manabu
Murali, D.
Murata, Toshihiro
Murkin, Andrew S.
Murphy, Cormac D.
Murru, Siva
Musabayane, C. T.
Musci, M.
Musiol, Robert
Mustafa, Mohd Rais
Muthusamy, Visalini
Muzart, Jacques
Nagai, Yoshinori
Nagasawa, Kazuo
Naha, Pratap C.
Naidoo, Yougasphree
Naila, Aishath
Naito, Yuji
Najahi, Ennaji
Najdanovic-Visak, Vesna
Nakaki, Toshio
Nakamura, Kazuhiko
Nakamura, Mkikio
Nakamura, Yoshimasa
Nakazawa, Jun
Namba, Kosuke
Nanjundaswamy, Ananda
Naota, Takeshi
Nardoni, S.
Nau, Werner
Navacchia, Maria Luisa
Navarrete-Vázquez, Gabriel
Naviglio, Silvio
Nawwar, M. A.
Nayak, Sukanta
Nazzaro, Filomena
Ndip, R. N.
Nechifor, Marioara
Neergheen, Vidushi
Negri, Giuseppina
Nehira, Tatsuo
Nerín, C.
Nesterov-Mueller, Alexander
Nevárez-Moorillón, Guadalupe Virginia
Neves, Graça
Das Neves, J.
Nezamzadeh-Ejhieh, Alireza
Ngouela, Silvère
Ni, Bukuo
Nicasio-Torres, María Del Pilar
Nicaud, Jean-Marc
Nicoletti, Ferdinando
Niedziałkowski, Paweł
Nihei, Ken-Ichi
Nikolova, Milena
Nindo, Caleb
Nishihara, Yasushi
Nishiwaki, Hisashi
Nishiwaki, Nagatoshi
Nishiyama, Yasuhiro
Nishizono, Naozumi
Niu, Zhenbin
Nkenke, Emeka
Nobili, Stefania
Nordén, Bengt
Norimoto, Hisayoshi
Nostro, Pierandrea Lo
Novellino, Ettore
Novo, Salvatore
Novotarskyi, Sergii
Ntalli, Nikoletta G.
Numata, Keiji
Nwaehujor, Chinaka
Nwe, Nitar
Nyokong, Tebello
O'connor, Kevin E.
O’Keefe, S. F.
Oancea, Elena
Obenland, David
Obora, Yasushi
Obushak, N. D.
Odell, Luke R.
Ogawa, Akiya
Ogihara, Jun
Oh, Dong-chan
Oh, Jiyoung
Oh, Sei-Ryang
Oh, Seikwan
Ohnishi, Masatoshi
Ohno, Sho
Ohshima, Hiroshi
Ojeda, Manuel
Ojika, Makoto
Oka, Natsuhisa
Okada, Yoshinori
Okada, Youji
Okazaki, Toshiro
Okun, Ilya M.
Okuyama, Ryuhei
Olafsdottir, Elin Soffia
Olajide, Olumayokun A.
Olar, Rodica
Oliveira, Beatriz P. P.
Oliveira, Rita De Cássia M.
Oliveira, Ronaldo Lopes
Olivier, Denise K.
Olmo, Esther Del
Oltra, J. Enrique
Omata, Kenji
Omori, Alvaro Takeo
Opletalova, Veronika
Orbán, Norbert
Ortega, Angel L.
Ortona, Elena
Orzechowski, Arkadiusz
Osakada, Kohtaro
Osanai, T.
Osheroff, Neil
Ośmiałowski, Borys
Osumi, Kenji
Otsuka, Hideaki
Overduin, Michael
Pace, Andrea
Pacifico, Severina
Pacios, Luis
Paczesny, Sophie
Padmaperuma, Asanga B.
Pagan, Rafael
Pagano, Bruno
Page, Tony
Pagenkopf, Brian L.
Paller, Channing
Palmans, Anja R. A.
Palmeri, Mark L.
Palomo, Claudio
Palop, Juan Antonio
Pan, Cheol-Ho
Pan, Min-Hsiun
Pan, Tai-Long
Pan, Y. J.
Panayiotidis, Mihalis I.
Pandanaboina, Sahitya Chetan
Pandey, Kailash C.
Panke, Sven
Panpranot, Joongjai
Pansare, Sunil V.
Pant, Archana
Panzella, Lucia
Papetti, Adele
Parameswaran, Narayanan
Paraskevopoulou, Adamantini
Pariani, Giorgio
Park, Il-Kwon
Park, Ji-Yeon
Park, S. H.
Park, So-Young
Park, Youngja
Parker, Kathlyn A.
Parker, William B.
Parkinson, Gary N.
Parodi, Brunella
Parvu, Marcel
Pasantes-Morales, H.
Paschke, Reinhard
Passamonti, Sabina
Passarella, Daniele
Pataj, Zoltan
Patrice, Vanelle
Paudyal, Bishnuhari
Paulsen, Berit Smestad
Pavan, Giovanni M.
Pavelić, Sandra Kraljević
Pawar, Rahul S.
Pawlaczyk, Izabela
Pecul, Magdalena
Pedraza-Chaverri, José
Pedrosa, Rozangela Curi
Pei, Dehua
Pei, Jian
Pelaez, José Walter
Pelegrín, Pablo
Pelkonen, Olavi
Pellegrino, Daniela
Pellizzon, Cláudia Helena
Peltzer, M.
Pena, Miren
Peng, Wen-Huang
Peng, Xiaohua
Penissi, Alicia B.
Penso, Michele
Perego, Carla
Pereira-Netto, Adaucto Bellarmino
Pereira, Leonel
Pereira, Olívia R.
Pereira, Romaiana
Pérez-Jiménez, Jara
Perez-Jorge, Conchita
Pérez-Lamela, Concepción
Pérez-Palacios, T.
Perez-Pinzon, M. A.
Pérez-Trujillo, Míriam
Pérez, Andy J.
Perez, J. J.
Pericàs, Miquel A.
Perrier, Aurélie
Perrin, Charles L
Perrone, Daniela
Perry, Nigel Brian
Perticaroli, Stefania
Peruzzini, Maurizio
Peters, Verena
Pethe, Stéphanie
Petitjean, Anne
Pianet, Isabelle
Piaz, Fabrizio Dal
Picariello, Gianluca
Pielichowska, Kinga
Pieroni, Marco
Pietro, Rosemeire C. L. R.
Pietrucci, Fabio
Piga, Rosaria
Pigge, F. Christopher
Pike, Victor W.
Pinho, Olivia
Pinna, Gérard A.
Pinto, Eugénia
Piotrowska, Dorota G.
Piovezan, Anna P.
Piwowarski, Jakub P.
Planas, Joana M.
Planas, Marta
Plasencia, Javier
Platas-Iglesias, Carlos
Plavec, Janez
Plażuk, Damian
Plech, Tomasz
Pletcher, Mathew T.
Plourde, Guy L.
Pluskota-Karwatka, Donata
Poblaciones, Maria J.
Poce, Giovanna
Podolak, Irma
Pohanka, Miroslav
Poinsot, Verena
Polepally, Prabhakar Reddy
Poleshchuk, Oleg
Pontoni, Ludovico
Popiołek, Łukasz
Poppe, László
Poriel, Cyril
Porte, Cinta
Porto, Ana Lúcia Figueiredo
Potter, P. M.
Poucheret, Patrick
Pour, Milan
Pourquier, P.
Powers, Joseph R.
Pozo-Bayón, Maria Angeles
Prado, Soizic
Praly, Jean-Pierre
Preuss, Kathryn E.
Price, Andrew J.
Price, Neil P. J.
Prieto, Alicia
Prins, Leonard
Proestos, Charalampos
Proschak, Ewgenij
Prosser, R. Scott
Prüß, Birgit M.
Przybylski, Cédric
Psomas, George
Pu, Jian-Xin
Pu, Min
Puerta, M. Carmen
Puglia, Carmelo
Pulici, Maurizio
Pyne, Stephen
Qandil, Amjad M.
Qi, Chenze
Qi, Hong
Qian, Mingxing
Qian, Wenyuan
Qiao, Jennifer
Qin, Bo
Qin, Chuanguang
Qin, Xuemei
Qiu, Bo
Qiu, Ming-hua
Qiu, Shengxiang
Queiroga, Felisbina Luisa
Queiroz, Maria-João R. P.
Quock, Raymond M.
Qvit, Nir
Raczyńska, E. D.
Rádl, Stanislav
Rafii, Michael S.
Ragno, Gaetano
Rainha, Nuno
Raja, Boobalan
Ramos-Organillo, Ángel
Ramos, Márcio Viana
Rampoldi, Luca
Rand, Thomas
Randazzo, Antonio
Rani, Sudheer D.
Rao, P. N. Praveen
Raposo, M. Manuela M.
Rasekh, Manoochehr
Raspotnig, Günther
Rathore, Rajendra
Ravu, Ranga Rao
Rawal, Viresh H.
Rawel, H. M.
Rawlings, Neil D.
Rawson, Ashish
Rawson, Jeremy
Ray, Ratna B.
Read De Alaniz, Javier
Read, Daniel S.
Reany, Ofer
Regel-Rosocka, Magdalena
Regnault-Roger, Catherine
Rehse, Steven J.
Reiser, Georg
Reiser, Oliver
Rejman, Dominik
Ren, Fazheng
Ren, Hongjun
Ren, Jian-guo
Ren, Ping
Reyes, Rosa Estrada
Reyniers, Marie-franoise
Ribeiro Cerqueira, Miguel Ângelo Parente
Ribeiro, Marcelo L.
Ricardo Da Silva, Jorge
Ricart, Susagna
Ricci, Alfredo
Rice, Joseph E.
Richardson, Sarah
Richomme, Pascal
Riedmaier, Irmgard
Rigano, Daniela
Rigo, Benoît
Rinaldi, A. C.
Ringkjøbing-Jensen, Malene
Rinner, Uwe
Rio, Alberto Del
Ríos, Cristóbal De Los
Rissanen, Matti P.
Rivera, Augusto
Riveros Galan, David Santiago
Rizzotto, Marcela
Robert, E.
Robert, François
Roberts, Bryan
Robles-Zepeda, Ramón Enrique
Robles, Alfonso
Robles, Eva Sánchez
Robson, Mark
Rockenbach, Ismael Ivan
Rodembusch, Fabiano S.
Rodiño, Paula
Rodrigues, Ana Lúcia S.
Rodríguez-Arcos, R.
Rodriguez-Lopez, Jose Neptuno
Rodriguez, Jaime
Rodriguez, Saleta Vazquez
Rohn, Sascha
Rojo, Javier
Rosa, Antonella
Rosa, Luiz H.
Rose, Devin
Roseiro, Luísa B.
Rosemeyer, Helmut
Rosengren, K. Johan
Rotondo, Archimede
Rouanet, Jean-Max
Roumy, Vincent
Rourke, Jonathan P.
Roy, René
Roy, Sharani
Rozentsveig, Igor B.
Rozi, Mohamed
Rubert, J.
Rubis, Blazej
Rudolph, U.
Ruffoni, Barbara
Ruiz-Azuara, Lena
Ruiz-Gómez, Miguel J.
Rupasinghe, H. P. Vasantha
Rupenthal, Ilva D.
Rurali, Riccardo
Russell, Mark
Russo, Gian Luigi
Russo, Nino
Ruzicka, Ales
Ruzza, Paolo
Rybakova, Yu S.
Ryckman, Kelli K.
Ryu, Jae-Sang
Saavedra, Juan M.
Sackus, Algirdas
Saczewski, Franciszek
Saeki, Tohru
Sagebiel, John C.
Sahin, Selin
Saiano, Filippo
Sainlos, Matthieu
Saito, Masaichi
Saito, Shinichi
Saito, Yoshihiro
Saitoh, Masao
Sajeva, Maurizio
Sakagami, H.
Sakata-Haga, Hiromi
Salas, Cristián
Salas, Juan M.
Salazar-Olivo, Luis A.
Sale, Stewart
Saleem, Mohammad
Salem, Abdelfattah Z. M.
Salgueiro, Lı´gia
Salles, Christian
Salvador, Marcos
Samoc, Marek
Sánchez Chávez, Esteban
Sánchez-Delgado, Roberto A.
Sánchez-Moreno, Manuel
Sánchez-Vioque, R.
Sanchez, L.
Sandel, Johan
Sandoval, Claudia
Sandow, Shaun L.
Sani, Marc-Antoine
Sanjay, Nigam
Sanna, Gavino
Santagati, Andrea
Santiago, Helton
Santos-Silva, Alice
Saraiva, Lúcia M. F. S.
Saravanan, M.
Sarbu, Coster
Sardella, Roccaldo
Sarkar, Biprajit
Sarkar, Ujjal
Sarver, Jeff
Sassaki, Guilherme L.
Sathe, Shridhar
Sato, Takaaki
Saturnino, Carmela
Sautreau, Asmita
Savaskan, Nic E.
Savoia, Diego
Sawaya, Alexandra
Schanze, Kirk
Schatz, Jurgen
Schenning, Albertus P. H. J.
Schild, Lorenz
Schiller, Stefan M.
Schleicher, Erwin D.
Schlettwein, Derck
Schmalz, Hans-Günther
Schmeda Hirschmann, Guillermo
Schmidt, Jürgen
Schmidt, Thomas J.
Schneider, Bernd
Schneider, Gyula
Schnermann, Martin
Scholz, Martin
Schomburg, Lutz
Schreiber, Angélica Zaninelli
Schrekker, Henri S.
Schüller, Christoph
Schulman, Ira
Schulte, Gunnar
Schultz, Carsten
Schulz, Margot
Schumacher, U.
Schuster, David I.
Schwalbe, Harald
Schwarz, Wolfgang
Schweizer, Frank
Sciarini, Lorena
Scilimati, Antonio
Scipione, Luigi
Scognamiglio, Monica
Scorziello, Antonella
Scott, Colin
Scott, Lincoln
Scotti, Luciana
Scovassi, Anna Ivana
Sebestik, Jaroslav
Secci, Daniela
Seifert, Karlheinz
Seifert, Roland
Seijas Vázquez, Julio
Seitz, Oliver
Selbo, PÅl K.
Selig, Philipp
Semple, Susan
Senel, Mehmet
Sener-Aki, Esin
Seo, Shigemi
Séraphin, D.
Serpersu, Engin H.
Serra, Stefano
Serralheiro, Maria Luísa M.
Serrano, Carmo
Serrano, María
Serroni, Scolastica
Settanni, L.
Seubert, John M.
Sextl, G.
Seyfert, Hans-Martin
Seymour, E. Mitchell
Shah, Kavita
Shahgaldian, Patrick
Shahidi, Sheila
Shaik, Ahmad Ali
Shaik, Sason
Shan, Wei-Guang
Shang, Ming-Ying
Shanmugam, K. T.
Shapiro, Nancy L.
Sharma, Dipali
Sharpless, Norman
Shcharbin, Dzmitry
She, Gaimei
Shecterle, Linda M.
Shen, Juan
Shen, Ya-Ching
Shen, Yue-Mao
Sheng, Chunquan
Sherry, A. Dean
Sheu, Jyh-Horng
Shi, Da-Qing
Shi, Fa-Nian
Shi, Gangrong
Shi, Qian
Shi, Xiangyang
Shi, Xiaodong
Shiau, Chung-Wai
Shibasaki, Masakatsu
Shieh, Dar-Bin
Shih, Ming-Der
Shih, Wen-Pin
Shikov, Alexander N.
Shilin, Chen
Shim, Soon-Mi
Shima, Seigo
Shimada, Ichio
Shimada, Tsutomu
Shimizu, Hiroki
Shimoda, Hiroshi
Shin, Hyung Shik
Shin, Sung Chul
Shinitzky, M.
Shipley, Paul
Shirota, Yasuhiko
Shoben, Abigail B.
Short, John D.
Shova, S.
Shoyama, Yukihiro
Shridhar V. Andurkar, Shridhar
Shum, Anderson Ho Cheung
Shuto, Tsuyoshi
Si, Ting
Siddiqi, Rahmanullah
Siddiquee, Tasneem A.
Siebert, Hans-Christian
Sievänen, Elina
Silva, Carlos M.
Silva, Daniel Varon
Silva, Filomena
Silva, Hélder Daniel
Silva, Jefferson Rocha A.
Silván, Jose M.
Silvestri, Romano
Simal-Gándara, Jesús
Simerska, Pavla
Simirgiotis, Mario
Simmonds, Monique S. J.
Simonneaux, Gerard
Simonsen, Henrik
Simpson, Garth
Singh, Ashutosh
Singh, Baljit
Singh, Itender
Singh, S. K.
Singh, Suresh B.
Singh, Udai P.
Siniscalco, Dario
Siodłak, Dawid
Sippl, Wolfgang
Sips, Patrick Y.
Siqueira, Ionara
Sironi, Maurizio
Sissi, Claudia
Sivakumar, Manickam
Skaltsa, Helen D.
Skrobik, Yoanna
Sladic, Dusan
Sławiński, Jarosław
Sliva, Daniel
Smart, Robert C.
Šmejkal, Karel
Smietana, Michael
Smith, Alan
Smith, Robert E.
Smoleński, Piotr
Soares, Luiz
Sobarzo-Sánchez, Eduardo
Sobczak, Izabela
Sobkowski, Michal
Soengas, Raquel
Sokolenko, Taras M.
Solfrizzo, M.
Soliman, Karam F. A.
Song, Baoan
Song, Jinhua
Song, Yang
Song, Yang-Heon
Song, Zhihong
Sooman, Linda
Sørlie, Morten
Soto, Carmen
Soukoulis, Christos
Soulère, Laurent
Sousa, Andreia F.
Sousa, Maria João
Souza, Lauro M.
Sozio, Piera
Sozzi, Gabriella
Spangenberg, Hans Christian
Sparr, C.
Spatafora, Carmela
Specht, Alexandre
Speer, Karl
Spina, Federica
Spiteller, M.
Sreelatha, S.
Sridhar, Radhakrishnan
Srivatsan, Eri S.
Stadler, Marc
Staerk, Dan
Stafford, Gary
Staines, Henry M.
Stamatatos, Theocharis C.
Stanisz, Beata
Stanković, Milan S.
Stark, Timo D.
Starks, Courtney M.
Steenkamp, Vanessa
Stefani, Hélio A.
Steinbrenner, Holger
Stephanidou-Stephanatou, Julia
Stevanato, Roberto
Stevenson, Philip C.
Stoddart, Fraser
Stojanovic, Ivana
Stolle, Achim
Stone-Elander, Sharon
Stone, Michael
Stonik, Valentin A.
Stoppacher, Norbert
Strano, Michael S.
Stratakis, Manolis
Stratmann, Johannes W.
Struga, Marta
Su, Shu-Jem
Suarez, Alirica
Subramani, Ramesh
Sugimoto, Keiichiro
Sugimura, Hideyuki
Sugumaran, Manickam
Suh, Hyung Joo
Sun, Daekyu
Sun, Haoran
Sun, Hui
Sun, Jingzhi
Sun, Kang
Sun, Ming-Liang
Sun, Peng
Sundberg, Richard
Sung, Ping-Jyun
Surup, Frank
Sussman, Fredy
Sutherland, J. B.
Sutthivaiyakit, Somyote
Suzuki, Hiroyoshi
Suzuki, Nobuaki
Suzuki, Yasuo
Svete, Jurij
Sypecka, Joanna
Szeląg, Małgorzata
Szewczyk, Nathaniel J.
Taatjes, Craig A.
Tabata, Jun
Taglialatela-Scafati, Orazio
Taglietti, Angelo
Tago, Megumi
Tahara, Hidetoshi
Tai, Guihua
Taïeb, David
Taioli, Simone
Takagi, Ryukichi
Takase, Masayoshi
Takasu, Kiyosei
Takeda, Kei
Takeda, Yoshiyu
Takemori, Hiroshi
Taketsugu, Tetsuya
Takimiya, Kazuo
Talbot, Jennifer
Talib, Wamidh H.
Talukdar, Ranajit K.
Tan, Nguan Soon
Tan, Yi
Tanabe, Yoo
Tanaka, Hitoshi
Tanaka, Masakazu
Tang, Chih-Hsin
Tang, Wei
Tang, Li
Tang, Xing
Tang, Yiqun
Tao, Feng
Tao, Lei
Tapia, Alejandro
Tapondjou, La
Tarasiuk, Jolanta
Taupitz, Matthias
Tauskela, Joe
Tava, Aldo
Tegoni, Matteo
Teixeira, Róbson
Tel-Zur, Noemi
Tempesta, S.
Teng, Chieh-Lin
Tepe, Jetze J.
Terasaka, Naoki
Terekhova, Irina V.
Terenzi, Hernán F.
Teschke, Rolf
Tesio, Franco
Testa, Ugo
Tezuka, Yasuhiro
Thevissen, Karin
Thiéry, Valérie
Thomas, Brian F.
Thomas, Christophe M.
Thomas, Eric
Thomas, Raymond H.
Thompson, Colin
Thompson, Kyle J.
Thomson, Regan J.
Timperio, Anna Maria
Titorenko, Vladimir I.
Tittmann, Kai
Tjandra, Nico
Tokunaga, Makoto
Tolstoy, Pete M.
Tomi, Félix
Tomic, Oliver
Tommonaro, Giuseppina
Tong, Meiping
Tonks, Nick K.
tori, Motoo
Torii, Seiji
Török, Marianna
Toropov, Andrey A.
Torrens, Francisco
Tortorano, Anna Maria
Toshiro, Noshita
Toth, Gabor
Touaibia, Mohamed
Tournier, Nicolas
Touzani, Rachid
Toyooka, Naoki
Tramontano, Enzo
Trehy, Michael L.
Trevisan, Gabriela
Trevitt, Adam
Trindade, Alexandre
Tron, Gian Cesare
Truhlar, Donlad
Tsai, Chia-Wen
Tsai, Li-Chu
Tsai, Shauwei
Tsai, Tung-Hu
Tsai, Yow-Fu
Tsalev, Dimiter
Tsanaktsidis, John
Tsatsaroni, E.
Tsay, Chien-Yie
Tsoleridis, Constantinos A.
Tsopmo, Apollinaire
Tsourkas, Andrew
Tsuboi, Yasuyuki
Tu, Tao
Tups, Alexander
Turel, Iztok
Tuzimski, Tomasz
Tuziuti, Toru
Tzakos, Andreas G.
Tzen, Jason Tc
Ueda, Kouhei
Ueda, Mitsuhiro
Ullah, Saif
Uquiche, Edgar
Urabe, Hirokazu
Urbanczyk-Lipkowska, Zofia
Usami, Yoshihide
Usenik, VAlentina
Utrilla, Pilar
Vacca, Rosa
Vaidyanathan, Ganesan
Vaira, Dino
Valencia, Gregorio
Valensin, Daniela
Valentao, Patrícia
Valentin, Eulogio
Valentini, Massimiliano
Valero, M.
Valledor, Annabel
Van Bogaert, I. N. A.
Van Dam, Mike
Van De Graaf, Stan F. J.
Van De Venter, Maryna
Van De Vijver, Pieter
Van Den Boorn, Jasper G.
Van Den Elsen, Jean Mh
Van Der Aa, Lieke
Van Dongen, Guus A. M. S.
Van Eijk, Marco
Van Hoek, Monique L.
Van Kaer, Luc
Van Lanen, Steven
Van Nhien, Albert Nguyen
Van Nieuwenhove, Carina P.
Van Ree, Teunis
Van Rij, Ronald,
Van Staden, Van
Van Valckenborgh, Els
Vandamme, Marc
Vanden Eynde, ean Jacques
Vanderlinden, Evelien
Vanetti, Maria Cristina Dantas
Vannacci, Alfredo
Vaquero, M. J. Rodríguez
Varese, Giovanna Cristina
Vargas-Baca, Ingacio
Vargas-Bello-Pérez, E.
Vargas-Rodriguez, Yalma L.
Varney, Veronica A.
Vasconcelos, Vitor
Vasilevich, Natalya I.
Vavříková, Eva
Vavvas, Demetrios G.
Vayssade, Muriel
Vaz, Maria G. F.
Vedani, Angelo
Vediyappan, Govindsamy
Veitch, Nigel C.
Veleirinho, Beatriz
Velez, Zélia
Velkov, Tony
Vennerstrom, Jonathan
Venturelli, Sascha
Verardo, Vito
Verbruggen, Alfons
Vermerris, Wilfred
Verrax, Julien
Vetter, Ingrid R.
Vetvicka, Vaclav
Viappiani, Cristiano
Vicario, Jose L.
Vicente, Gonzalo
Vicente, José
Vidal, Hilario
Vieira, Ceferina
Vieira, Ivo José Curcino
Vieira, Margarida C.
Vieira, Nirton
Vilas-Boas, Miguel
Vilegas, Wagner
Villa, Federica
Villegas, Alejandro Madrid
Villmann, Carmen
Vinsova, Jarmila
Virag, Jitka A. I.
Viricelle, J.-P.
Viruel, Maria Angeles
Vitali, Luca
Vitalini, Sara
Vitorović-Todorović, Maja D.
Vivier, Melané A.
Vlashi, Erina
Vlastos, Dimitris
Vock, Carsten
Voituriez, Arnaud
Volf, Petr
Volpatti, Donatella
Volpi, Nicola
von Recum, H. A.
Vosjan, Maria
Vougioukalakis, Georgios C.
Vriens, Joris
Vuorela, Pia
Wadsak, W.
Wagner, Carl E.
Wähälä, Kristiina
Wahlqvist, Mark L.
Wakamatsu, Hideaki
Wakshlag, Joseph
Waksmundzka-Hajnos, Monika
Walsby, Charles J.
Walseth, Timothy F.
Walz, Riger
Wan, Hayley
Wan, Jian-Bo
Wan, Meng-Wei
Wanderley, Almir G.
Wang, Changchun
Wang, Changguan
Wang, Chi Chiu
Wang, Chih Kuang
Wang, Ching-Chiung
Wang, Chun-Ru
Wang, Chung-Yi
Wang, Cunde
Wang, Feng
Wang, Fuyi
Wang, Guowei
Wang, Hao-Yang
Wang, Hexiang
Wang, Hongbing
Wang, Hui-Min David
Wang, Jeffrey
Wang, Jeh-Jeng
Wang, Jian-Wu
Wang, Jigang
Wang, Jinchuang
Wang, Kai-Yun
Wang, Leyong
Wang, Limin
Wang, Meng-Jiy
Wang, Peng George
Wang, Richard Y.
Wang, Shenguang
Wang, Shengyang
Wang, Tzu-Pin
Wang, Wei-Hsien
Wang, Wen
Wang, Wen-Jwu
Wang, Xi Cun
Wang, Xianbao
Wang, Xianzhong
Wang, Xiaowu
Wang, Xinkun
Wang, Xunsi
Wang, Y.
Wang, Yan-Qing
Wang, Ying
Wang, Yingyao
Wang, Yingying
Wang, Yonghua
Wang, Zhanguo
Wang, Zhanzhong
Wang, Zhijun
Waring, Michael
Waser, Mario
Watanabe, Naoharu
Waters, Stephen P.
Wawra, Stephan
Weghe, Pierre Van De
Wei, Jian-He
Wei, Jianhe
Weisman, Gary
Welch, K. D.
Wells, Timothy N. C.
Welsh, William J.
Wertz, Philip
Wesolowski, Marek
West, Caroline
White, Alex
Whitfield, Dennis M.
Wildhalm, Michael
Willars, Gary
Willför, Stefan M.
Williams, David E.
Williams, Ffrancon
Williams, Ian H.
Willner, Itamar
Wills, Martin
Wilson, Lee D.
Wilson, W. David
Wimmer, Zdenek
Windt, Michael
Wingfield, Paul T.
Wingler, Kirstin
Winiarska, Katarzyna
Wondrak, Georg T.
Wong, Chi Chun
Wong, Chi-Huey
Wong, Harvey
Wong, Henry N. C.
Wong, P. K.
Wong, Wai-Yeung
Wong, Wallace
Woo, Mi Hee
Wood, Michael R.
Wood, W. Gibson
Wood, Warren J. L.
Woods, Mark
Worth, Randall G.
Wrackmeyer, Bernd
Wrenger, Carsten
Wright, Aaron T.
Wright, Dennis
Wu, Alan Hb
Wu, Albert M.
Wu, Bin
Wu, Chieh-Hsi
Wu, Chih-Hsiung
Wu, Chin-Chung
Wu, Christine D.
Wu, Chun-Chi
Wu, Defeng
Wu, Ho-Shing
Wu, Horng Cherng
Wu, Huayue
Wu, Huiquan
Wu, Jie
Wu, Jin-Yi
Wu, Li-Chen
Wu, Ming-Jiuan
Wu, Pao-Chu
Wu, Peng
Wu, Qiong
Wu, Shih-Hsiung
Wu, Shihua
Wu, Shu-Jing
Wu, Tian-Xiang
Wu, Tzi-Yi
Wu, Tzong-Yuan
Wu, Xiao-Feng
Wu, Yang-Chang
Wu, Yong
Wu, Zhaoxin
Wuest, Frank
Xi, Chuanwu
Xi, Zhen
Xiang, Lan
Xiao, Dequan
Xiao, Qiang
Xiao, Wei-Lei
Xie, Haihui
Xie, Pei-Shan
Xiong, Bing
Xiong, Fei
Xodo, Luigi E.
Xu, Baoshan
Xu, Changjie
Xu, Chunlin
Xu, Huajian
Xu, Jian-Hong
Xu, Li-Wen
Xu, Shichao
Xu, Xudong
Xu, Zhenbo
Xue, Hui
Xue, Ming
Xue, Ying
Yadav, Hariom
Yadav, Madhav
Yaegaki, Ken
Yagi, Kiyohito
Yalcin, D.
Yamada, Hiroko
Yamada, Shizuka
Yamagishi, M.
Yamaguchi, Masahiko
Yamaguchi, Yoshiki
Yamaki, Koji
Yamamoto, Norio
Yamamoto, Yasunori
Yamamoto, Yoshinori
Yamato, Takehiko
Yamauchi, Satoshi
Yamauchi, Takahiro
Yamazaki, Shigekazu
Yamazaki, Takashi
Yanada, Reiko
Yanagita, Ryo C.
Yang, Bao
Yang, Changqing
Yang, Chi-Chiang
Yang, Guangzhong
Yang, Hsin-Ling
Yang, Jian
Yang, Kun-Lin
Yang, Li
Yang, Ling
Yang, Liqun
Yang, Liuqing
Yang, Meihua
Yang, S. C.
Yang, Shun-Fa
Yang, Te-Fang
Yang, Wen-Chin
Yang, Xiao-Hua
Yang, Xichuan
Yang, Ying-Wei
Yang, Yuedong
Yang, Yuh-Shyong
Yang, Z.-Q.
Yang, Zhijun
Yao, Ching Fa
Yao, Guangmin
Yao, Jianhua
Yao, Mei-Cun
Yao, Ping
Yatsunyk, Liliya
Ye, Song
Ye, Xingqian
Ye, Zhengfang
Yen, Tzu-Chen
Yenjai, Chavi
Yeo, Siok-Koon
Yeung, Bryan K. S.
Yeung, Ying-Yeung
Yew, Wen Shan
Yim, Yong-Hyeon
Yli-Kauhaluoma, Jari
Yokoyama, Yoshihito
Yoo, Eun-Sook
Yoo, Hyuk Sand
Yoon, Do Yeung
Yoon, Do-Young
Yoon, Juyoung
Yoshida, Hiroto
Yoshida, Takashi
Yoshimatsu, Mitsuhiro
Yoshimi, Yasuharu
Youn, Buhyun
Yu, Chu-Yi
Yu, Chung-Shan
Yu, Jianmei
Yu, Steve S.-F.
Yu, Wen-Bin
Yuan, C. S.
Yuan, Lujiang
Yuan, Qipeng
Yuan, Weizhong
Yuan, Zhong
Yuanjiang, Pan
Yuasa, Hideya
Yuasa, Junpei
Yuen, John Wm
Yuk, Hyun-Gyun
Yusa, Shin-Ichi
Zabaras, Dimitrios
Zacchetti, Daniele
Zacchino, Susana
Zaharia, Valentin
Zalapa, Juan E.
Zalapa, Juan Ernesto
Zamani, Akram
Zamora-Ros, Raul
Zandi-Sohani, N.
Zangara, Andrea
Zaprutko, Lucjusz
Zarrelli, Armando
Zaug, Joe
Zech, S. G.
Zeng, Aiwu
Zeng, Dexing
Zeng, Minfeng
Zeng, Su
Zhang, Guojian
Zhang, Han-Ting
Zhang, Hanqi
Zhang, Ji-Lin
Zhang, Jianjun
Zhang, Jun
Zhang, Lian-Feng
Zhang, Liangren
Zhang, Lu-Yong
Zhang, Pei-Cheng
Zhang, Pengfei
Zhang, Qinghai
Zhang, Weiyun
Zhang, Wen
Zhang, Wenbing
Zhang, Wenchang
Zhang, Xiao-Mei
Zhang, Xiaokun
Zhang, Xiuli
Zhang, Xiurong
Zhang, Yan-Bo
Zhang, Yang
Zhang, Yansheng
Zhang, Yin
Zhang, Yingjun
Zhang, Yu
Zhao, Chun-Xia
Zhao, Chunshun
Zhao, Haifeng
Zhao, Hong
Zhao, Hua
Zhao, Jianzhang
Zhao, Jing
Zhao, Ming
Zhao, Mouming
Zhao, Peng
Zhao, Qinshi
Zhao, Yongxi
Zhao, youxin
Zheng, Li
Zheng, Qi-Huang
Zheng, Wei
Zhong, Yu-Wu
Zhou, Dong-mei
Zhou, Guangxiong
Zhou, Guohua
Zhou, Hong
Zhou, Huiping
Zhou, Li-Gang
Zhou, Pei
Zhou, Xue-Rong
Zhou, Yifa
Zhu, Duanwei
Zhu, Hai-Liang
Zhu, Hongjun
Zhu, Jianglong
Zhu, Nanwen
Zhu, Qin-Yu
Zhu, Wei-Ming
Zhu, Weihong
Zhu, Ying
Zhuang, Xiuli
Zia, Muhammad
Zielinska, Danuta
Zieliński, Henryk
Zimmerman, Steven C.
Żołnowska, Beata
Zondlo, Neal J.
Zong, M. H.
Zong, Xu-Xiao
Zorc, Branka
Zou, Chenyu
Zou, Yong
Zou, Zhongmei
Zoumpoulakis, Panagiotis
Zrenner, Rita
Kochi, Takuya

